# Fungal Biodiversity of the Most Common Types of Polish Soil in a Long-Term Microplot Experiment

**DOI:** 10.3389/fmicb.2019.00006

**Published:** 2019-01-22

**Authors:** Jarosław Grządziel, Anna Gałązka

**Affiliations:** Department of Agricultural Microbiology, Institute of Soil Science and Plant Cultivation – State Research Institute, Puławy, Poland

**Keywords:** soil metagenomics, fungal genetic biodiversity, microplot experiment, sequencing, pH

## Abstract

The aim of the study was to investigate fungal genetic diversity in eight different types of soil in a long-term microplot experiment founded in 1881 in Puławy, Poland. The experiment consists of eight plots (14 m^2^), each 1 m deep with concrete walls, filled with profiles of different soils. The soils represent the most common Polish soil types (Cambic Leptosol, Fluvic Cambisol, Gleyic Chernozem, Cambisol and Haplic Cambisol, two Brunic Arenosols and Haplic Luvisol). Each soil was characterized by different pH (from 4.0 to 7.5) and organic carbon content (4.5–21.3 g kg^-1^). The soil structure was not destroyed by compaction because the soils had always been cultivated by hand. The same plant species were always grown in all plots at the same time and the soils received the same fertilization. Moreover, the soils were always under the same weather conditions. *Ascomycota* was the most abundant phylum in all samples, ranging from 70 to 90% of total fungi. Some genera (*Mortierella*, *Solicoccozyma*, and *Mycosphaerella*) were found to be adapted to a wide range of pH. Acidic soils were dominated by *Talaromyces*, *Cladophialophora*, *Devriesia*, and *Saitozyma*, while good quality soils primarily consisted of *Plectosphaerella*, *Tetracladium*, and *Mortierella*. The study confirmed previous reports that pH has a decisive influence on soil fungal diversity, but also indicated the strong impact of soil type itself. These studies have launched a new cycle of research in these historical soil profiles.

## Introduction

The biological component of soil is extremely important for the maintenance of soil health (Doran and Zeiss, 2000). The microbial component of the soil is responsible for a myriad of functions (80–90% of processes in soil are reactions mediated by microbes), including soil humus formation, cycling of nutrients, degradation of xenobiotics, improvement of soil structure, and effects on plant health (Nannipieri et al., 2003). The structural organization of soil particles and soil pores provides a spatially heterogeneous habitat for microorganisms, characterized by different substrate, nutrient and oxygen concentrations, water contents, as well as variable pH values (Sessitsch et al., 2001).

In the available literature, we can find many definitions of soil quality that take into account various parameters (physical, chemical, microbiological, and biochemical). The most common is the definition that “soil quality indexes could be defined as the minimum set of parameters that, when interrelated, provide numerical data on the capacity of a soil to carry out one or more functions” (Acton and Padbury, 1994). So far, no universal formula has been developed to allow precise measurements of soil quality, especially for agricultural soils. The reason for this may be the lack of standardization of many methods, and heterogeneity of soil and natural conditions (vegetation as well as climate). Different statistical methods are used to establish soil quality in agricultural soils. These methods are focused on biotic and abiotic indicators. In the literature, we can find indexes constructed exclusively by enzyme activities such as: enzyme activity number (EAN), biological index of fertility (BIF), and soil alteration index. The EAN index contains the sum of four enzymes: dehydrogenase, phosphatase, protease, and amylase (Beck, 1984). The Soil Alteration index contains the sum of seven enzymes: arylsulphatase, β-glucosidase, phosphatase, urease, invertase, dehydrogenase, and phenoloxidase (Puglisi et al., 2006). For most indexes, dehydrogenases are the dominant group of enzymes in the assessment of soil quality. In addition, many parameters are used to evaluate soil quality, including pH, microbial biomass, and organic matter content. Recently, molecular and genetic methods have also been included (Bastida et al., 2008). The results from DNA analysis could be useful, not only for determining the structure of microorganism communities, but also for determining what functions they perform. These analyses should also include a taxonomic examination of the fungi in agricultural soils. The continually evolving genetic studies and metagenomics will probably soon lead to the development of such indicators in soil quality assessment.

In recent years metagenomics revolutionized the knowledge of microorganisms inhabiting different environments, including soil. It allowed identification of non-culturable microbes and their metabolic capacities. These data contributed to the expansion of the existing “tree of life,” shedding more light on the evolution of living organisms (Hug et al., 2016; Tedersoo et al., 2017). Most of the metagenomic studies focus on bacterial diversity (Sengupta and Dick, 2015; Eo and Park, 2016; Fernandez et al., 2016), although fungal-bacteria ratio is often high in soil ecosystems in which fungi contribute more than 50% to the total biomass (Ananyeva et al., 2006).

Czaban et al. (2010), when studying the soils of their experimental object (founded in 1881), found relationships between CFU (colony-forming unit) numbers of various microbial populations and the volumes of soil pores. Especially interesting was the strong relationship between indexes of the soil fungal communities, presenting the relationships among hydrophilic, xerotolerant and xerophilic fungi, and the mean size of the soil pore. According to another study, at pH 4.5, a significant increase in the fungi-bacteria ratio and a general microbial activity decline was observed. This effect may be caused by, among other things, the releasing of free aluminum in the soil, which occurs at about pH 5.0. The second observation was strong inhibition of plant growth, which decreases the availability of organic root-derived carbon for the bacteria (Aciego Pietri and Brookes, 2008).

**Figure 1:**
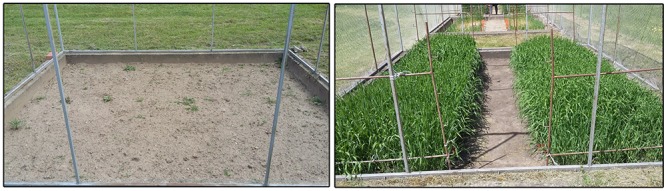
Example of the experimental microplots, before sowing (left), spring (right).

The aim of this study was to investigate differences in the fungal microbiome composition in eight different types of soil that are the most common soil types in Poland. The study was conducted on microplots (Figure 1), that were founded in 1881. The advantage of using microplots is the equal cultivation of each soil and exposure to the same weather conditions. The microplots include good quality soils (as Gleyic Chernozem, Fluvic Cambisol, Cambic Leptosol, Cambisol (Eutric), Haplic Luvisol, as well as acidic, poor quality soils: Brunic Arenosol I, Brunic Arenosol II, and Haplic Cambisol.

Until now, microplots have been used for studies to determine the influence of soil type on crop yields, measurement of the total number of microorganisms (culture-dependent techniques), and also to determine physicochemical parameters of soils, such size of soil particles and soil porosity (Table 1). This study is the first approach to determine the composition of fungi by a culture-independent method; will allow us to monitor changes in microbial composition over the years, starting from 2016.

**Table 1:** Historical studies on soils collected in microplots founded in 1881 in Puławy.

Studied parameters	Date (of experiment)	Reference
Total number of culturable bacteria and fungi	1994	Martyniuk, 1997
*Pseudomonas* abundance		
Biomass		
Soil organic matter		
Dehydrogenases and phosphatases activities		
Plant productivity		
Plant productivity	2006	Sułek, 2010
Size of soil particles	2007	Czaban et al., 2010
Volume of soil pores		
Porosity		
Water content		
pH		
Organic matter		
Total number of culturable fungi and bacteria		
*Penicillium* abundance		
Fungi xerotolerance		
pH	1994, 2015	Siebielec et al., 2015
Soil organic matter		
Soil available phosphorus, potassium, magnesium		
Total number of culturable fungi		
*Azotobacter* abundance		
*Actinomycetes* abundance		
Dehydrogenases and phosphatases activity		
16S NGS sequencing (total bacteria abundance)	2016	Grządziel and Gałązka, 2017
Dehydrogenases activity		
pH		
Soil functional analysis – Biolog EcoPlate		

The removal of variables, such as location, different effects of wind, and other factors, allowed us to determine the impact of soil type, its structure, and characteristics on microorganisms’ ability to colonize and survive. Our research hypothesis assumes that, depending on the type of soil, the fungal communities will differ across soil types, and secondly, the buffering capacity of soil will promote the maintenance of a stable microbial composition over the years.

## Materials and Methods

### Study Site

The study was performed at the microplot facility at the Institute of Soil Science and Plant Cultivation – State Research Institute in Puławy, Poland (GPS coordinates: 51.415218 and 21.960489). The experimental blocks consisted of eight soils of different origin (Table 2). Three soils were characterized by high organic carbon, pH 7.3–7.5 and good quality for plant cultivation: Cambic Leptosol, Fluvic Cambisol, and Gleyic Chernozem. Another three soils were acidic (pH 4.0–4.7), characterized by low organic carbon, and poor quality for cultivation: Haplic Cambisol and two Brunic Arenosols (I, II). The last two soils, Haplic Luvisol and Cambisol (Eutric), exhibited low organic carbon, pH 5.4–5.6 and relatively good conditions for cultivation of certain plants. The short history and description of the microplot experiment is available in Supplementary Material S1.

**Table 2:** Types of soil used in the experiment.

Soil type (current names)	Soil names (historical)	pH	C_org_	DHa
Brunic Arenosol (I)	Acidic brown soil/Dystric Cambisol	4.0	4.57	7.47
Brunic Arenosol (II)	Acidic brown soil/Dystric Cambisol	4.5	7.53	0.99
Haplic Cambisol (Dystric)	Dystric Cambisol/typical brown soil	4.7	5.27	2.98
Haplic Luvisol	Brown soil developed from loess/Eutric Cambisol	5.4	6.87	35.73
Cambisol (Eutric)	Typical brown soil/Eutric Cambisol	5.6	7.67	60.03
Cambic Leptosol	Rendzina	7.3	11.0	112.27
Gleyic Chernozem	Black-earth/Phaeozem	7.4	21.3	117.97
Fluvic Cambisol	Brown alluvial soil/Eutric fluvisol	7.5	8.2	49.88

*C_*org*_, organic carbon (g kg^-*1*^); DHa, dehydrogenases activity (μg g^-*1*^ dry soil), pH was measured in water. Each parameter was measured in 2016, historical measurements can be found in (Siebielec et al., 2015)*.

All 8 soils were under the same weather conditions and hand tillage management. The same plants were cultivated at the same time in each microplot (barley was the most recent crop).

Samples of bulk soil were collected in April 2016, from 15 representative spots per soil type, at a depth of 0–20 cm. Subsequently, soil samples were pooled, sieved through a 2 mm sieve, and stored at -20°C for future analysis.

Since 1979, the plots have been planted mostly with cereals as the main crop with mineral fertilizers, and as the second crop, mainly mustard, phacelia, or leguminous plants. In 1984, the plots were fertilized with compost (80 t ha^-1^) under potatoes (Czaban et al., 2010).

### Total DNA Extraction, PCR, and Sequencing

Fresh soil samples were weighed, and 300–350 mg were collected into 1.5 mL tubes for extracting DNA with FastDNA^TM^ SPIN Kit for Soil (MP Biomedical), according to manufacturer’s instruction. Purity and concentration were measured with a NanoDrop 1000 Spectrophotometer (Thermo Fisher Scientific). DNA was diluted with sterile Milli-Q water to 10 ng μl^-1^ concentration and sequenced at Genomed S.A. (Warsaw, Poland) in 2 bp × 250 bp paired-end technology using the Illumina MiSeq system. Amplification of the hypervariable ITS1 region was performed with Q5 Hot Start High-Fidelity 2× Master Mix accordingly to the manufacturer’s instruction with ITS1Fl2 and 5.8S primers (Schmidt et al., 2013). Authors are aware that there are more specific primers available and since the ITS1 region introduces more bias through unequal lengths of the amplicons, only forward reads were taken for the analysis. Raw FastQ files (both forward and reverse) were deposited in the NCBI SRA database (Table 3).

**Table 3:** Summary of deposited raw sequences in fastq format.

Soil type	Sample identification
Gleyic Chernozem	SAMN08474026
Fluvic Cambisol	SAMN08474025
Cambic Leptosol	SAMN08474024
Cambisol (Eutric)	SAMN08474029
Haplic Luvisol	SAMN08474027
Haplic Cambisol	SAMN08474023
Brunic Arenosol II	SAMN08474030
Brunic Arenosol I	SAMN08474028

*Public data is available in the NCBI Sequence Read Archive (SRA) database (https://www.ncbi.nlm.nih.gov/sra/SRP132335)*.

### Biolog^®^ FF Plates^TM^ (Biolog Inc., Hayward, CA, United States)

One gram of each soil was suspended in 99 mL of sterile water and vortex for 20 min at room temperature. The suspension was left to settle for 30 min at 4°C (Weber and Legge, 2009). Each well of was inoculated by 120 μl of suspension and incubated at 25°C for 7 days in the OmniLog^®^ ID System multiplate reader (Biolog Inc., Hayward, CA, United States). The intensity of the wells color development was collected every 30 min by the built-in reading camera and saved as OmniLog units (referred in this paper as OU), generated by the Biolog^®^ OmniLog PM software. The plates were prepared in triplicates.

### Statistical and Bioinformatics Analyses

#### DADA2 R Package – Resolving Amplicon Sequence Variants (ASVs)

Progress in metagenomics and metataxonomics has improved bioinformatics analysis. To date, the most common first stage is the development of OTUs (operational taxonomic units), which cluster reads based on their identity, usually at 97%. With the increased availability of high-performance computers, ASVs (amplicon sequence variants), also called exact sequence variants, are increasingly used and recommended. Alongside single-nucleotide resolution and accuracy, this method allows us to compare independent experiments at any time. More details and accuracy benchmarks of ASV resolving, using DADA2 software are available (Callahan et al., 2016).

Taking into account possible biases related to unequal lengths of ITS1 regions, ASVs were resolved only from forward reads using DADA2 version 1.6 package (Callahan et al., 2016) in R version 3.4.3 (R Core Team, 2016) with the following parameters: FastQ files were delivered demultiplexed and adapters were removed by Genomed S.A. Next steps were conducted in our department.

Subsequently in DADA2, using *filterAndTrim* and based on quality plots, sequences were trimmed to 220 bp, the first-left 25 bp were removed (containing primers and low quality bases). Filtering of sequences was set to: *maxN* = 0, *maxEE* = 2, *truncQ* = 2, where *maxN* is maximum number “N” bases, *maxEE* corresponds to maximum expected errors calculated from quality score [EE = sum (10^-Q/10^)] and *truncQ* parameter truncate reads at the first instance of a quality score less than or equal to 2. Other parameters were set to default. The error rates were estimated by *learnErrors*, where n-reads were set to 10^6^. Sequences were dereplicated using *derepFastq* with default parameters and exact sequence variants were resolved using *dada*. Next *removeBimeraDenovo* was used to remove chimeric sequences, applying the consensus method. From a total of 19640 unique, dereplicated sequences, 93 were identified as chimeras (∼0.005%) and removed from the sequences table.

#### DADA2 R Package – Classifying Sequences Against Reference Dataset

Taxonomy was assigned against the latest version of UNITE database (7.2 version, built 2017.12.01) with the dynamic clustering thresholds, using Naïve Bayesian Classifier (Wang et al., 2007) implemented in *assignTaxonomy*. This step assigns reads to six taxonomy levels (from phylum to the genus). The function *assignSpecies* provides exact matching to the species level, but in this study we considered the genus level as the most accurate for short (250 bp) amplicon sequencing technology. The minimum, maximum, and mean sample read count after sequence re-replication were, respectively: 210334, 279454, and 230583. The full classification (from the Kingdom to the Genus) is included in the Supplementary Table S6.

#### Phyloseq R Package – Data Curation

The resulting taxonomy and reads-count tables constructed in DADA2 were appropriately converted and imported into the phyloseq (1.22.3) package (McMurdie and Holmes, 2013). The first step was to remove all taxa other than Fungi (Plantae, Chromista, Rhizaria, Stramenopila, Alveolata, Protista, and Metazoa), which in total, represented about 4%. After this step, the minimum, maximum, and mean sample read count were, respectively: 180294, 270292, and 219170.9. Reads were then rarefied, setting the seed to 10000 (making this step reproducible) and new samples size as 180294. After rarefying, 37 “OTUs” were removed, because they were no longer present after random subsampling.

#### Phyloseq R Package – Alpha Diversity

**Figure 2:**
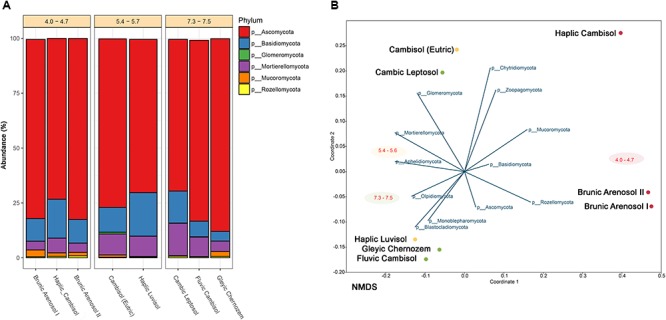
**(A)** Classification of fungal community at the phylum level corresponding to each soil in terms of relative abundance. Soils sharing similar pH are grouped. Unclassified reads and phyla below 1% are substracted. **(B)** NMDS (Non-metric Multidimensional Scaling) analysis of soil samples correlated to each fungal phyla abundance.

Different alpha diversity measures were calculated using the *phyloseq* package. Observed diversity and Shannon index for each sample are illustrated in Figure 2. More indexes (Chao1, ACE, Simpson, Fisher) can be found in Supplementary Table S1.

#### Vegan R Package – PERMANOVA

The rarefied data was used to calculate if soil type and its pH were significantly correlated to fungi composition. For this purpose, Bray-Curtis distance calculation was applied and permutation was set to 999 and PERMANOVA was calculated using the *vegan* R package (Oksanen et al., 2013).

#### SEED2 – Local BLAST and Species Hypothesis DOIs Assignment

To ensure future reproducibility of the results, it is wise to generate a unique DOI number which is always the same for the same nucleotide sequence. Even if fungal taxonomy changes, a detailed picture of exactly what species were found in previous experiments will still be possible. For this purpose each of ASVs from DADA2 was additionally classified against UNITE database (7.2) using local BLAST implemented into SEED2 software^1^. The following parameters were used: *E*-value 10^-5^, similarity minimum 99%, coverage minimum 90%. From 19547 sequences, 869 were successfully assigned to the different species hypothesis (SH). Next for each SH a Digital Object Identifier (DOI) was assigned from the UNITE analysis tool^2^. This document can be found in Supplementary Table S2.

#### PAST – Non-metric Dimensional Scaling (NMDS)

PAST software (Hammer et al., 2001) was used for NMDS applying the Bray-Curtis distance matrix calculations.

#### phyloT and iTOL – Phylogenetic Tree Generation

**Figure 3:**
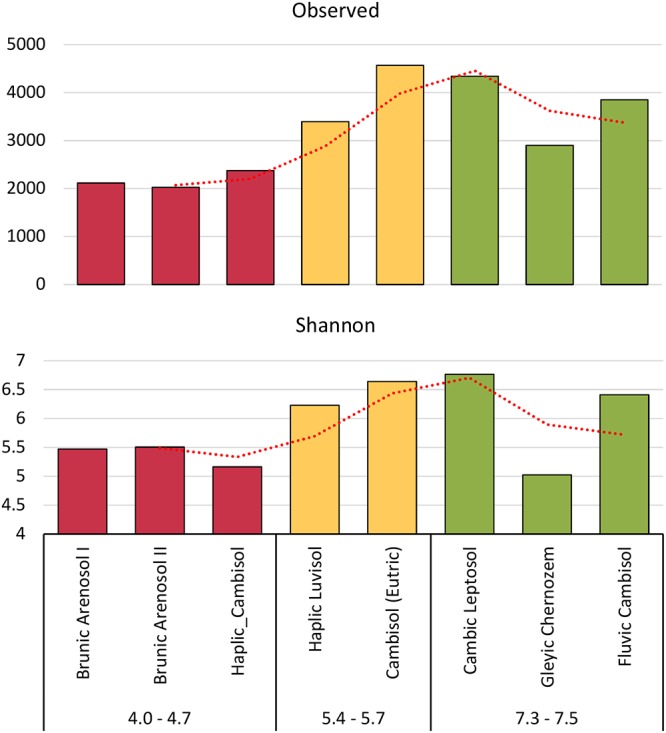
Shannon’s H’ and observed biodiversity indexes of fungal communities, based on total Amplicon Sequence Variants (ASVs).

The phylogenetic tree (Figure 3) was prepared using two online tools. First, phyloT^3^ was used to generate a tree in the newick format, based on the first 50 most abundant genera. Secondly the iTOL^4^ was utilized to annotate chosen features. The soil samples were divided into three groups, based on the pH values.

#### Biolog^®^ – Statistical Analysis

The data calculations were performed in the R-software, version 3.5.0 (R Core Team, 2016). The background color from water (A01 well) was subtracted from the readings of all wells at each time point. Results from triplicates were averaged. The results were transformed to AWCD (average well color development) by dividing the OU of all wells (excluding water) by 95 (Garland and Mills, 1991). Readings were selected at a point of 120 h. After that point, the color intensity of most wells started to decrease and by that time the color intensity had reached a plateau. The AWCD of each sample was analyzed by Tukey HSD *post hoc* test. Additionally, the AWCD was calculated for each group of substrates (carbohydrates, amino acids, amines and amides, carboxylic acids, and polymers), the classification of substrates was based on (Zak et al., 1994). The second calculated parameter was the area under the curve (AUC), using the trapezium rule (Aho et al., 2018), which better reflects the substrate utilization in time, taking into account not only the intensity of the color at the maximum point but also the kinetics of the reaction (Preston-Mafham et al., 2002).

#### Principal Component Analysis (PCA)

The PCA analysis was performed using the *ggfortify* package (Tang et al., 2016). All data was scale-transformed. Details and statistics of PCA calculation can be found in Supplementary Table S3. The data for Biolog^®^ (FF plates) is obtained from this work, following parameters: AWCD of Biolog^®^ (ECO plates), dehydrogenases activity and organic carbon content are obtained from the authors previous work (Grządziel and Gałązka, 2017), and the mean pore size indexes can be found in (Czaban et al., 2010).

## Results

### Fungal Diversity in Different Types of Soil

The collected soils, which belong to different types, despite over 130 years of cultivation, constitute a very valuable and relatively stable experimental object. Studies conducted by other researchers suggested that even after 20 years, the content of bioavailable elements and organic matter of soils remained at a similar level. Using next generation sequencing, it is possible to determine the composition and variety of fungi, which in the future, can serve as bioindicators of soil quality and condition. Almost 450 fungal genera were classified (from 157 in Brunic Arenosol I to 223 in Haplic Luvisol), belonging to 14 different phyla. The most common phylum (Figure 2A) among collected soils is Ascomycota, representing 70–90%. The second most abundant is Basidiomycota (4–20%). Mortierellomycota constitutes 4–15%. The other fungi jointly comprise from 1 to 4%. NMDS analysis (Figure 2B) revealed, that the most dominant fungi (at the Phylum level) have no clear preference for pH or soil type. However, such correlation can be observed among less numerous fungi, such as Rozellomycota (0.2–0.9%), showing a higher prevalence in acidic soils. It has already been demonstrated that some of the fungi belonging to Rozellomycota prefer soils with extreme pH values, but it has been suggested that the presence of eukaryotic hosts, for whom these fungi are obligatory pathogens, may be of greater importance (Tedersoo et al., 2017). Glomeromycota have been detected almost exclusively in good-quality, pH-neutral, and slightly acidic soils, but are close to non-existent in acidic soils (below 0.01%). Their occurrence is probably related to a more developed root system in soils with good agricultural suitability, where plant growth is more extensive, as Glomeromycota are mainly mycorrhizal fungi, establishing symbiosis with 85–90% of plants (Gałązka et al., 2017).

Biodiversity indexes were calculated on the basis of all ASVs, not only those correctly classified in the UNITE database. Rare taxa and taxa that are not known to date have been taken into account. Both the Shannon’s index (H’) and observed biodiversity, have shown that the biodiversity of fungi is correlated with the pH of the soil (Figure 3). The other indicators are available in the supplement. (S1). The lowest Shannon’s index was shown for highly acidic soil (H’ = 5.2–5.5). Soils of pH 5.4–5.6 had H’ = 6.2–6.6, while, the best quality soils had H’ = 5.0–6.8. The most surprising was the decline in the biodiversity index in the Gleyic Chernozem, which has a pH 7.4 and is the best quality soil for plant cultivation with the highest organic carbon content.

A study on bacterial biodiversity has shown one of the highest biodiversity values for this soil (Grządziel and Gałązka, 2017), which can be explained by the fact that bacteria fill up the niche in this soil to a greater extent. In studies on the relationship between the physicochemical properties of soils and the presence of microorganisms (Czaban et al., 2010), it has been shown that Gleyic Chernozem has the highest number of bacterial CFU (colony forming units) among all eight soils, with the lowest number of fungal CFU, consistent with this thesis.

**Figure 4:**
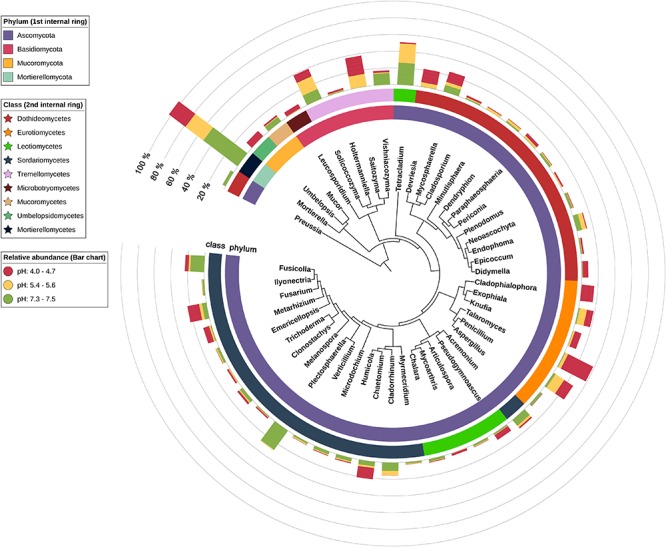
Phylogenetic tree of the 50 most abundant fungi. First internal ring’s coloring indicates phylum, second – class and bar chart colors indicate pH group of each soil.

The results from PERMANOVA revealed that fungi genera diversity is strongly pH-dependent (*P* = 0.008, *F* = 2.26, 999 permutations) and showed no clear soil type dependency. The occurrence of fungi correlated with soil pH is very well visible at the genus level (Figure 4). We can therefore divide the identified fungi into three groups. The first group is represented by genera present irrespective of pH and soil type, such as *Mortierella* (7–23% of total classified genera), *Solicoccozyma* (3–10%), *Mycosphaerella* (0.2–11%), *Fusarium* (1–8%), *Penicillium* (0.4–6%). The second group consists of fungi that prefer neutral or slightly acidic pH, e.g., *Tetracladium*, *Cladorrhinum*, *Plectosphaerella*. The third group includes fungi found almost exclusively in strongly acidified soils: *Saitozyma*, *Devriesia*, *Didymella*, *Cladophialophora*, and *Talaromyces*. The most acidic soil (Brunic Arenosol I, pH 4.0) is dominated by *Talaromyces*.

**Figure 5:**
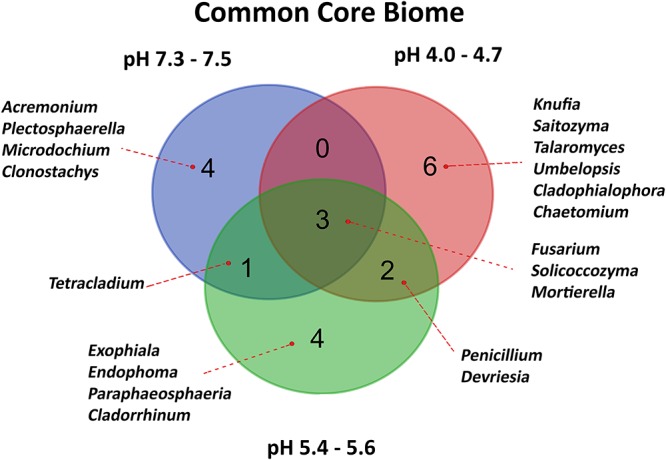
Venn diagram representing Common Core Biome of fungal communities characteristic of different soil pH groups. Fungal 20 representatives were selected, assuming minimum abundance equal to 1% and the presence in all soils of a pH group.

For each pH-related soil group, core fungi were calculated, assuming minimum abundance equal to 1% and requirement that given genus is present in all soils of a group. As a result, 20 different genera were selected, of which only three (Figure 5) were common for all soils: *Fusarium*, *Solicoccozyma*, and *Mortierella*. The most acidic soil group is then exclusively represented by six genera: *Knufia*, *Saitozyma*, *Talaromyces*, *Umbelopsis*, *Cladophialophora*, and *Chaetomium*. The core fungi of the highest quality soils are *Acremonium*, *Plectosphaerella*, *Microdochium*, and *Clonostachys*. Another four genera are exclusively present as a core biome in two slightly acidic soils: *Exophiala*, *Endophoma*, *Paraphaeosphaeria*, and *Cladorrhinum*.

### Biolog^®^ FF Plates

**Figure 6:**
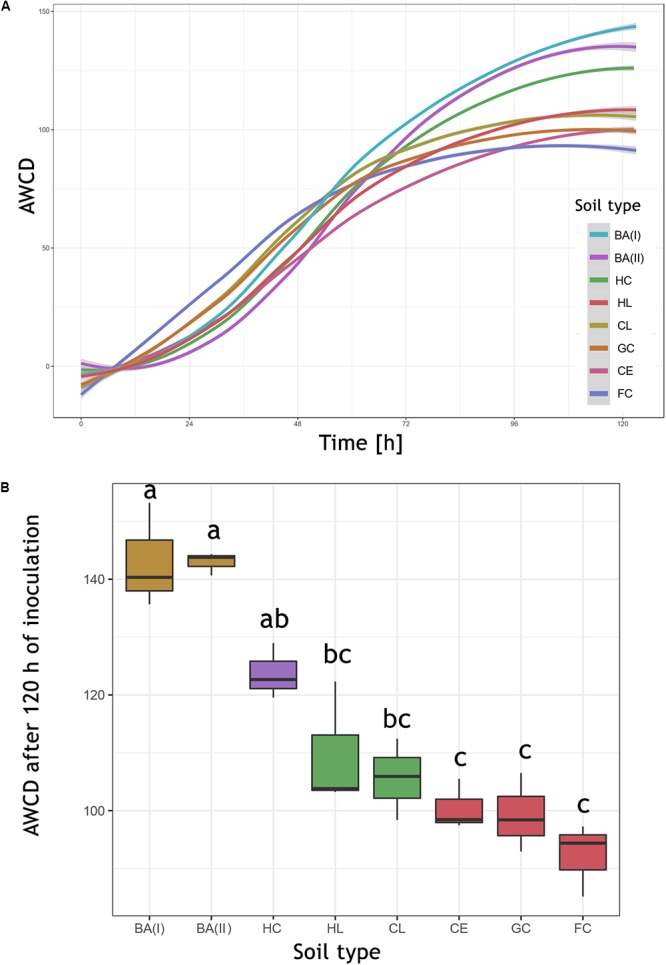
The AWCD (average well color development) expressed in Biolog^®^ OmniLog Units. **(A)** Kinetics of the mean AWCD from replicates (*n* = 3). **(B)** AWCD at 120 h, boxplot illustrating the median of AWCD (horizontal line), upper and lower quartile (corresponding borders of the boxplot), significant differences calculated by Tukey HSD test are indicated by letters. HC, Haplic Cambisol; BA (I, II), Brunic Arenosols I and II; HL, Haplic Luvisol; CL, Cambic Leptosol; GC, Gleyic Chernozem; CE, Cambisol (Eutric); FC, Fluvic Cambisol.

The Biolog^®^ FF plates were originally developed for the analysis of individual fungi and their identification, but they are also successfully used for the study of fungal populations in various environments (Borowik et al., 2017). The kinetics of AWCD (Figure 6A) showed a different rate of total metabolism for each soil. In the first hours of incubation (0–48 h) a slightly more intensive metabolism can be observed in eutrophic soils (of 5.94%). At the end of the measurement period, the highest intensity of color development was observed in the dystrophic soils (29.48% more comparing to eutrophic soils). The differences were statistically significant and according to the Tukey HSD test (Figure 6B), AWCD at 120 h grouped soils of similar type and pH. All AWCD measurements (from 0 to 120 h) can be found in Supplementary Table S4.

**Figure 7:**
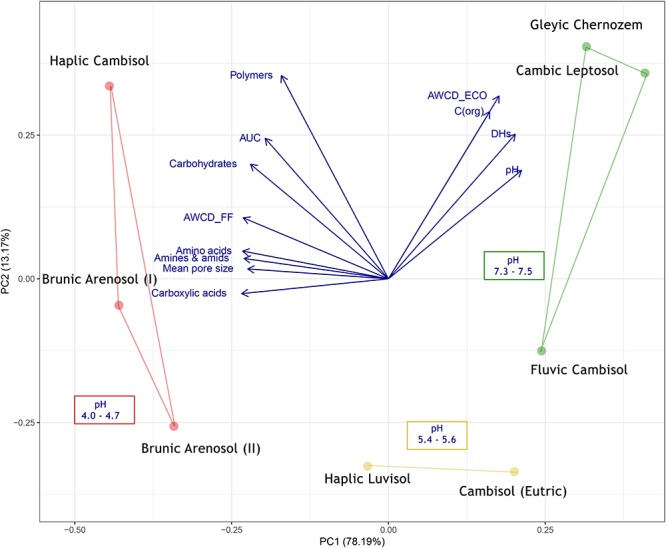
PCA (Principal Component Analysis) of soil 11 parameters.

Combining the results from this work with another parameters: mean pore size [from Czaban et al. (2010) paper], AWCD from ECO plates, organic carbon content and dehydrogenases activity [from Grządziel and Gałązka (2017) paper] the PCA analysis was conducted (Figure 7).

The first conclusion from the analysis is the opposite level of metabolism of bacteria (ECO) and fungi (FF), which may indicate different niches (e.g., soil pores) occupied by these organisms. The highest level of fungal metabolism was related to dystrophic soils, of poor quality, contrary to bacteria, that were most active in the eutrophic soils. The mean pore size is also positively correlated with acidic, dystrophic soils, as well as with the high level of metabolism of individual substrate groups, which is consistent with the results obtained by Czaban et al. (2010), although his methods were limited to cultivable microorganisms and soil samples were taken almost 10 years ago (in 2007). The AUC was also positively correlated to dystrophic soils, especially to Haplic Cambisol. The correlation between AUC and AWCD in different time points indicated, that the results from 120 h was chosen adequately (0.861, *P* = 0.006). The similarly high correlation was observed for 96 h (0.893, *P* = 0.003), and 72 h (0.944, *P* = 0.0004), but not for earlier time points, e.g., 48 h (0.270, *P* = 0.518), 24 h (-0.242, *P* = 0.564). The abovementioned calculation is included in Supplementary Table S5.

## Discussion

The microplot experiment was established in 1881, making it one of the oldest in Europe. The combination of different soils in a small area allows precise measurements, which are independent of weather conditions or agrotechnical treatments. One of the concerns about this type of research is the likelihood of soil degradation due to the lack of access to primary parent rocks and water sources that occurred in the places where they originated. In both, historical and current studies it was repeatedly examined and confirmed, that these microplots are valuable experimental objects, enabling the understanding of the effects of soil type on plant yields (Martyniuk, 1997; Sułek, 2010), providing information on the relationship between fungi occurrence, soil pore sizes, and other physical parameters (Czaban et al., 2010). Some studies demonstrated that the soil studied in this experiment has retained values such as pH, organic carbon content, bioavailable potassium, phosphorus or magnesium forms, at nearly a constant level for more than 20 years, indicating a high buffer capacity of the soil (Siebielec et al., 2015).

One of the most recent studies on this experimental object was the analysis of a bacterial microbiome using NGS sequencing (16S rRNA region) and functional analyses using the Biolog^®^ system on ECO plates (Grządziel and Gałązka, 2017). The authors confirmed that the composition of bacteria is closely correlated with the type of soil, but primarily with the soil pH. Eutrophic soils are characterized by the greater biodiversity of bacteria (Shannon index), as well as the higher activity of dehydrogenases and general metabolism (Biolog^®^, ECO). Conversely to the bacteria, the current study on fungal composition has shown that the metabolic activity of fungi was negatively correlated with soil quality and pH (Figure 7). The dystrophic soils were characterized by a lower rate of fungal biodiversity than the eutrophic soils of good quality, but at the same time, the utilization of substrates (Biolog^®^, FF plates) was more intensive in the dystrophic soils. This shows that not only alpha-diversity and the number of individual species are important for soil health, but above all, the functional biodiversity that these microorganisms provide makes the ecosystem function efficiently and sustainably, as it was stated by (Frac et al., 2018).

The most abundant phyla in each soil were Ascomycota (70–88%) and Basidiomycota (4–20%) which is consistent with other researches on different soils, for example forest soils (Buée et al., 2009), with a predominance of Basidiomycota or tundra soils (Schadt et al., 2003), where Ascomycota dominated. The third most abundant phylum was Mortierellomycota (4–15%) represented only by the genus *Mortierella* sp. which was also found in a relatively high number in forest soils (Buée et al., 2009). *Mortierella* sp. is known as saprobic and ubiquitous, as is transported by a wind and rain. An increasing number of studies concern its ability solubilize phosphorus, as well as its usefulness in increasing crop yields and in establishing symbiosis with agricultural plants (Fröhlich-Nowoisky et al., 2015). In our study, *Mortierella* sp. did not show any significant preference for soil quality or pH, accounting for 6–11% in acidic soils, 13–15% in eutrophic soils, except for Cambic Leptosol, where it was 23%. Interestingly, some of the fungi commonly described as dominant in many soils have shown low prevalence in our studies. One example is *Trichoderma* sp., which is considered to be one of the most common fungi in nature due to the high-stress tolerance and rapid growth rate (Hagn et al., 2003; Pagano et al., 2017), in our research was present in only 0.06–1%. Similarly, the *Fusarium* sp., considered as dominant in most of the soils (Wakelin et al., 2008), here represented between 1 and 4.5% in seven of the eight soils, exceptionally in the Haplic Cambisol 8%. On the other hand *Solicoccozyma* sp. is commonly found in soils with a high salt content (Mokhtarnejad et al., 2016; Zajc et al., 2017) in our microplots accounted from 2 to 9%.

It has been confirmed repeatedly that one of the most important factors affecting the composition of fungal communities in the soil is the pH, e.g. (Zhang et al., 2016), but it is also noted that most fungi are endemic, their biodiversity comparisons should be more local than global (Frac et al., 2018), that is why global comparisons between fungi abundances data sometimes do not correlate with each other.

Modern experimental methods as NGS or functional analysis (e.g., Biolog^®^) allow to generate a lot of useful data in a relatively short time, so to learn the composition of hundreds of different species of bacteria or fungi and also insight into the metabolic profile of entire communities. The authors are aware of the limitations of modern methods (incomplete reference databases, the problem with reproducibility of results caused by dynamic changes in soil). Methods that are now considered outdated or even historic also had their advantages and disadvantages. The research conducted by (Czaban et al., 2010) on the correlation of the mean soil pore size and soil fungi diversity was based mainly on culture methods. However, in very general terms, these results correlate very well with those obtained today. For example, his results suggested that the mean size of soil pore is positively correlated with a fungi abundance, and the fungi CFU (colony forming units) are negatively correlated with bacterial CFU number. He also stated that acidic soils promote the growth of the fungi, due to the higher tolerance to the hydrogen ions, conversely to the most of bacteria. In this study, we proved that those relations are still valid (Figure 7), more specifically, mean soil pore size and a low soil pH is positively correlated to total fungi metabolism and negatively correlated to the bacteria abundance and its activity.

Referring to another research conducted on this experimental site and based on classical methods (Siebielec et al., 2015) investigated the occurrence of bacteria and fungi and concluded that the most numerous bacteria were found in the Gleyic Chernozem and Fluvic Cambisol, while in dystrophic and acidic soils, the total number of bacteria was several dozen times smaller. The highest number of fungi was found in acidic soils (Brunic Arenosol I and II). When we compare these results with the modern methods used in this work, the overall conclusions are similar. What is interesting and contradicts Siebielec’s work is that the number of bacteria *Azotobacter* sp., referring to his results, was relatively high in soils with pH above 5.6, while the results based on sequencing (Grządziel and Gałązka, 2017) did not indicate their presence in any of the soils. The authors are not sure whether these differences result from the fact that depending only on culture methods, a classification error was made (based only on bacteria’s ability to atmospheric nitrogen fixation), or whether the relative amount of *Azotobacter* sp. in soils was insufficient to be detected by the NGS or a third possibility, short (250 bp) fragments of the 16S rRNA gene were classified into a different genus due to a possible high genetic similarity.

These studies have shown that microbial communities are closely linked to the soil type, which has been repeatedly proven in studies in various regions of the world. There was a strong division of soils where the metabolic potential of fungi is significant (dystrophic soils; Cambisol Chapel, Brunic Arenosols) while bacteria metabolism is relatively low and those in which, despite greater biodiversity (e.g., Shannon’s H Index), the total metabolism was relatively decreased (eutrophic soils: Gleyic Chernozem, Fluvic Cambisol, Cambic Leptosol, Haplic Luvisol, Cambisol). The results obtained by modern methods (NGS, Biologists ECO/FF plates) were also correlated with historical research, conducted, among others, on the basis of individual colony counting, cultivation of bacteria, morphological identification.

## Author Contributions

AG contributed to design of the experiments, statistical analysis, and manuscript writing and revision. JG contributed to experimentation, manuscript writing, and bioinformatics analysis. JG and AG contributed to data interpretation and manuscript preparation.

## Conflict of Interest Statement

The authors declare that the research was conducted in the absence of any commercial or financial relationships that could be construed as a potential conflict of interest.

## Footnotes

1 http://www.biomed.cas.cz/mbu/lbwrf/seed/

2 https://unite.ut.ee/analysis.php

3 https://phylot.biobyte.de/

4 https://itol.embl.de/

## Supplementary Material

TABLE S1 Alpha diversity indexes calculated for each of eight soil fungal community.

TABLE S2 Species hypothesis DOI’s.

TABLE S3 PCA calculations.

TABLE S4 Biolog FF AWCD.

TABLE S5 Biolog FF AUC.

TABLE S6 ITS classifications (UNITE).

MATERIAL S1 Short history and description of microplot experiment.
