# Supplementary material for: Fungal Biodiversity of the Most Common Types of Polish Soil in a Long-Term Microplot Experiment
Source: Front Microbiol. 2019 Jan 22;10:6. doi: 10.3389/fmicb.2019.00006 (PMC6357946; doi:10.3389/fmicb.2019.00006)
Supplement: MATERIAL S1 — Short history and description of microplot experiment. [file Data_Sheet_1.docx]

| **Supplementary material: The historical microplot experiment – a short history and description** |
| --- |

The microplot experiment, as the one of the oldest experiment in the Europe, was originally established in 1881 in Pulawy, Poland. The very first descriptions and manuscripts were written in Russian language, since Pulawy was a part of the Russian Empire, named “Nova Alexandria”, and the online versions are not available. One of the first published information about the experiment was the 1896 study „Historical note about the construction of experimental plots at Institute of Nova Alexandria” also written in Russian. Another two references were written in Polish making them not easily available to the general audience:

1. Strzemski M., Historia Gleboznawstwa Polskiego, Powszechne Wydawnictwo Rolnicze i Leśne, 1980, p. 172 **(in Polish)**,
2. Siebielec, G.; Siebielec, S.; Podolska, G. Comparison of microbial and chemical characteristics of soil types after over 100 years of cereal production. Polish J. Agron. 2015, 23, 88–100 **(in Polish)**.

The microplot experiment was founded by Konstanty Malewski and Grzegorz Rudinskij. They built seven (now there is eight) microplots by digging holes of 12 m^2^ each and 1 m deep.

**

**

**Fig. S1 Source: http://historiaiung.pulawy.pl/Biogramy.php)**

The walls were bricked up, while the bottom was not concreted. The substrate on which the soils were placed was sterile sand buried on the original soil. Seven different soil types, characteristic to Poland, were collected in a few locations in Pulawy and its surrounding villages (e.g. Konskowola, Kazimierz Dolny, Pozog, Kepa Pulawska).


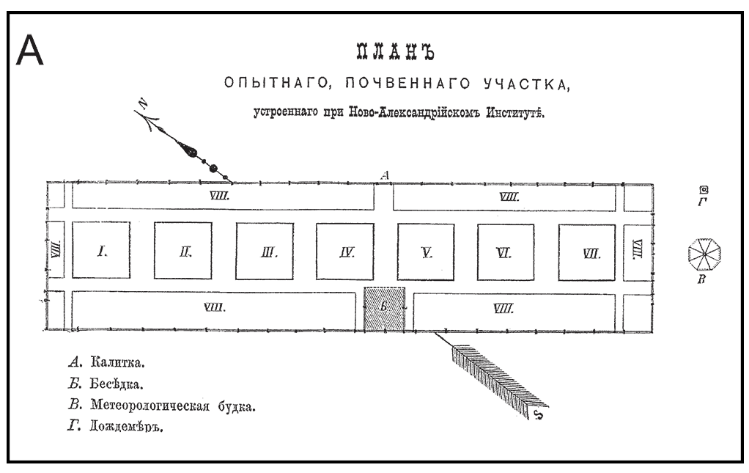


**Fig. S2** Schematic map of microplot experiment established in 1881. The map was originally published in 1896 in a study „Historical note about the construction of experimental plots at Institute of Nova Alexandria” written in Russian, not available online.

The soil profiles were collected without destroying the soil structure, preserving the natural soil horizon layers. From the beginning of the experiment, all soils were treated equally including the same cultivated plant, agriculture management (hand tillage), irrigation and fertilization.


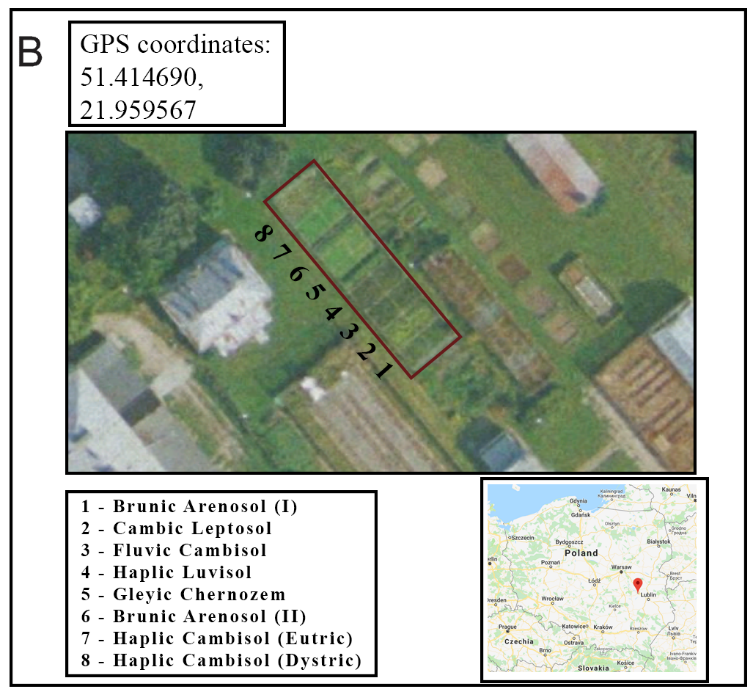


**Fig. S3** The current view of the microplot experiment (source: Google Maps) with GPS coordinates and soil types indication.

Different soil types collected in the microplot experiment represent the most typical and common soils found in Poland. They differ in physicochemical parameters, pH, organic carbon content and different textures. Every couple years these parameters as well as soil typing is repeated to monitor soil stability and ensure that soil types have not changed significantly over time.

The most current data is presented in the table below, including soil types, texture, pH, organic carbon content and dehydrogenases activity.

**Table S1** Soils used in experiment

| Soil type  (current names) | Abbreviation | Granulometric group / Soil Texture  (USDA classification) | pH | C_org_ | DHa |
| --- | --- | --- | --- | --- | --- |
| Brunic Arenosol (I) | BA (I) | loamy sand | 4.0 | 4.57 | 7.47 |
| Cambic Leptosol | CL | sandy loam | 7.3 | 11.0 | 112.27 |
| Fluvic Cambisol | FC | sandy loam | 7.5 | 8.2 | 49.88 |
| Haplic Luvisol | HL | fine sandy loam | 5.4 | 6.87 | 35.73 |
| Gleyic Chernozem | GC | fine sandy loam | 7.4 | 21.3 | 117.97 |
| Brunic Arenosol (II) | BA (II) | loamy sand | 4.7 | 5.27 | 2.98 |
| Cambisol (Eutric) | CE | medium sandy loam | 5.6 | 7.67 | 60.03 |
| Haplic Cambisol (Dystric) | HC | fine sandy loam | 4.5 | 7.53 | 0.99 |

The three of the collected soils represent acidic, dystric soils characterized by a low pH 4.0 – 4.7 (BA(I), BA(II), HC) . Another three soils are considered as a good quality group (CL, FC, GC), characterized by a pH 7.3 – 7.5 and the highest organic carbon content. The last two soils represents intermediate parameters and pH values 5.4 – 5.6 (HL, CE).

The main advantage of this microplot experiment is that all soils are collected in the very small area, and are subjected to the same conditions (geographical location, weather, the same climate etc.). It ensures that the data collected by using different methods (enzymatic activity, molecular techniques, substrates utilization etc.) depends only on the soil type and its characteristic.
